# How much will linked deformable registrations decrease the quality of multi-atlas segmentation fusions?

**DOI:** 10.1186/s13014-014-0251-1

**Published:** 2014-12-20

**Authors:** Carl Sjöberg, Silvia Johansson, Anders Ahnesjö

**Affiliations:** Section for Medical Radiation Physics, Department of Radiology, Oncology and Radiation Sciences, Uppsala University, Akademiska Sjukhuset, Sjukhusfysik Ing. 82, SE-751 85 Uppsala, Sweden; Elekta Instrument AB, Box 1704, S-75147 Uppsala, Sweden; Section for Oncology, Department of Radiology, Oncology and Radiation Sciences, Uppsala University, Akademiska Sjukhuset, Sjukhusfysik Ing. 82, SE-751 85 Uppsala, Sweden

## Abstract

**Background and purpose:**

Multi-atlas segmentation can yield better results than single atlas segmentation, but practical applications are limited by long calculation times for deformable registration. To shorten the calculation time pre-calculated registrations of atlases could be linked via a single atlas registered in runtime to the current patient. The primary purpose of this work is to investigate and quantify segmentation quality changes introduced by such linked registrations. We also determine the optimal parameters for fusing linked multi-atlas labels using probabilistic weighted fusion.

**Material and methods:**

Computed tomography images of 10 head and neck cancer patients were used as atlases, with parotid glands, submandibular glands, the mandible and lymph node levels II-IV segmented by an experienced radiation oncologist following published consensus guidelines. The change in segmentation quality scored by Dice similarity coefficient (*DSC*) for linking free-form deformable registrations, modeled by B-splines, was investigated for both single- and multi-atlas label fusion by using a leave-one-out approach.

**Results:**

The median decrease of the *DSC* was in the range 2.8% to 8.4% compared to direct registrations for all structures while reducing the computer calculation time to that of a single deformable registration. Linking several registrations showed a *DSC* decrease almost linear to the number of links, suggesting that extrapolation to zero links provides an observer independent measure of the inherent precision with which the segmentation guidelines can be applied.

**Conclusions:**

Linking pre-made registrations of multiple atlases via a runtime registration of a single atlas provides a feasible method for reducing computation time in multi-atlas registration.

## Introduction

Image segmentation for outlining targets and risk organs is a tedious and time consuming part of the radiotherapy process. Segmentation uncertainty can also contribute significantly to the total uncertainty in radiotherapy [1]. Atlas-based segmentation, where deformable image registration is used to transform segmentations from pre-segmented images, can be used both to reduce manual labor time and to decrease inter-operator variability [2,3]. Multi-atlas registration with label fusion, where the results from several independently registered atlases are automatically fused to yield a segmentation proposal, has been demonstrated to increase atlas based segmentation performance as compared to using a single atlas [4-6]. Several methods have been developed for the label fusion process, see e.g. [4,7].

A practical problem for implementing multi-atlas methods into clinical routine is the long calculation times needed for the deformable registrations of multiple image series. Several methods have been proposed in the literature to overcome this problem. One is to carefully choose and limit the number of individual segmentation proposals used in the fusion process [5,8]. It has also been proposed to select a subset of images based on their similarity and only register those deemed to most likely give a good final segmentation result [8,9]. The selection could be made initially, or after a fast registration using an affine transformation model with only a few degrees of freedom. Another approach would be to store pre-calculated transformations to a representative atlas that can be registered to the patient and used as a link of the premade registrations to the actual patient images. This would only be feasible if the segmentation quality losses caused by the linking itself are not too large. In a clinical setting, a large number of previously treated patients could potentially be of interest to use as multi-atlas material for new patients. With a very large atlas database available, pre-calculated deformable registrations could be clustered in a hierarchical strategy through strategically selected atlases serving as “hubs” to save computation time. Under such circumstances some atlas structures could be subject to several linked transformations which motivates to quantify the expected change in resulting segmentation quality as function of the number of applied linked transformations.

In this work we investigate the change in segmentation quality by comparing linked atlas registrations to direct registrations for structures relevant to radiotherapy of head and neck cancer. The result is determined for both individual segmentations and fused segmentation, where in the latter case we use a method based on probabilistic averaging of distance functions [10]. We also optimize the parameters of this fusion method for the set of head and neck atlases used in this work.

## Methods and materials

In atlas-based segmentation, a *moving* image *M*(*x*, *y*, *z*) is registered to a *fixed* image *F*(*x*, *y*, *z*) yielding a spatial transformation *T*_*M* ← *F*_(*x*, *y*, *z*) relating the positions of structures in *F* with the corresponding positions in *M*. Applying a transformation on a moving image results in a *registered* image *R*_*M* ← *F*_(*x*, *y*, *z*) = *M*(*T*_*M* ← *F*_(*x*, *y*, *z*)). To explicitly calculate *R*, interpolation must be used as in general grid point locations for the voxel positions in one of the images is by *T*_*M* ← *F*_(*x*, *y*, *z*) related to off-grid points in the other image. The structures in *M* are represented as a label map *L*_*M*_(*x*, *y*, *z*) which is transformed using *T*_*M* ← *F*_(*x*, *y*, *z*) such that voxels belonging to the same object are given the same label in the transformed image. In multi-atlas segmentation, the label images from several deformable registrations of different atlas images are combined in a label fusion process to provide the final segmentation proposal, as further described in section Label fusion.

Registrations can be *linked* by composing several transformations. A resulting transformation is then formed by *T*_*M* ← *I*(*l*) ← *F*_(*x*, *y*, *z*) = *T*_*M* ← *I*_(*T*_*I* ← *F*_(*x*, *y*, *z*)), or shorter *T*_*M* ← *I*(*l*) ← *F*_ = *T*_*M* ← *I*_ ∘ *T*_*I* ← *F*_ which is calculated by linking registration results from registering *M* via one or several intermediate images *I* and where *l* indicates the number of intermediate transformations that are used in the linking process, e.g. ${T}_{M\leftarrow I(2)\leftarrow F}={T}_{M\leftarrow {I}_2}\circ {T}_{I_2\leftarrow {I}_1}\circ {T}_{I_1\leftarrow F}.$ As the transformations *T*_*I* ← *F*_ for any given *F* and *I* can be pre-calculated and retrieved from a database, critical computer time savings can be achieved as compared to making the multiple registrations directly.

We will investigate the difference in segmentation quality using a leave-one-out evaluation strategy with a set of atlases for targets and risk organs in head and neck radiotherapy. The atlases consist of planning computed tomography image sets *A*_*i*_, *i* = 1, …, *N* for *N* = 10 atlas patients consistently segmented by an experienced radiation-oncologist following the guidelines of Lengele *et al.* [11]. The patients used as atlases were randomly selected from a database of radiotherapy patients treated for tumors in the head and neck region, so none of the patients had a completely normal anatomy. To the best of our knowledge, no specific guidelines are published for the node positive neck so we used the guideline for the node-negative neck for all segmentations. The structures used for comparison were medulla, mandible and right and left parotid glands, submandibular glands and lymph node regions II-IV. The lymph node regions were segmented as one contiguous structure per side.

Applying the leave-one-out approach, an atlas from the database is selected to represent a new patient image to which the remaining atlases can be registered. This yields for *N* = 10 a total of 90 pre-calculated registrations ${T}_{A_j\leftarrow {A}_i},i\ne j,$ which in the remainder of this work will be denoted with *T*_*j* ← *i*_ for simplicity. In the deformable registration process only the image information, and not the segmentations available, was used as to mimic clinical conditions. The segmentations transformed from the other atlases were then compared to the original, manually made segmentations for evaluation of the segmentation quality. The decrease in segmentation quality by using linked registrations compared to direct registration was assessed for both individual and fused segmentation proposals. We used the Dice similarity coefficient [12]

1 $$ DSC\left({B}_1,{B}_2\right)=2\frac{\left|{B}_1\cap {B}_2\right|}{\left|{B}_1\right|+\left|{B}_2\right|} $$

to score the segmentation qualities for the binary image volumes *B*_1_ and *B*_2_. This measure achieves a value of one for identical segmentations and zero for segmentations with no spatial overlap.

Another measure used was a fractional mean absolute distance measure (*fMAD*), calculated as the fraction of surface voxels for *B*_1_ within a given distance (in 3D) from the closest surface voxel of *B*_2_. A surface voxels for a binary volume is in this case defined as any object voxel sharing one or more sides, i.e. is 6-connected, to a background voxel. This gives an asymmetric distance measure describing how large fraction of a binary volume that is within some distance from a reference binary volume.

### Registration method

Each explicit registration was performed in two parts, first an affine registration which optimized parameters for translation, rotation, scaling and shearing. This transformation was then used as initialization for a deformable registration step modeled by B-splines [13]. This method parameterizes the transformation as a linear combination of compactly supported splines placed on a regular grid. The coefficients for the linear combination were optimized with regard to an image similarity measure. For this work, normalized mutual Information (*NMI*) [14] was used as the similarity measure.

All registrations were performed in a multi-resolution fashion using a Gaussian pyramid with four levels to reduce the risk that the optimization converges to a local optimum. A registration starts at the coarsest level, and the resulting transformations from each level are then used as initialization for the next level. This process was repeated until the final image resolution was reached. As the resolutions of the images are anisotropic with a slice thickness larger than the in-slice pixel sides, the down-sampling for the first level was only performed in the in-slice direction, and for the remaining levels the images were down-sampled with an equal factor for all dimensions. The value of the down-sampling scaling factor was 2 for all dimensions and levels. For the deformable registrations, the B-spline grid spacing was 8 mm in all directions for the finest resolution and down-sampled in accordance with the scheme outlined above.

As optimization method we used the adaptive stochastic gradient descent (ASGD) [15], as implemented in the Elastix package [16]. This method speeds up the registration process by using a sub-sampled set of intensity values for the computation of the image similarity measure and its derivatives. We used 2048 pairs of intensity values as the sub-sampled set, sampled at randomly selected locations within the image volumes. To achieve convergence of the stochastic optimization, the step size was reduced for each iteration with a fixed number of iterations calculated for each registration level. We calculated 500 iterations per level, which gave reasonable computation times for the individual registrations. Linear interpolation was used during registration and 3:rd degree B-spline interpolation for applying the final transformation to the moving image.

### Ethical approval

This work was approved by the Regional Ethical Review Board in Uppsala (2013/277).

### Linking of registrations

**Figure 1:**
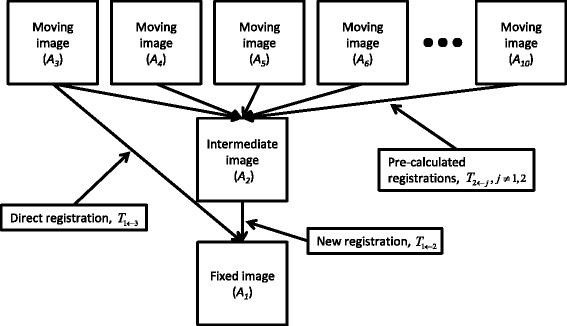
**Illustration of registration linking.** Segmentation transfers for the case of *i =* 1, *k =* 2 and *N =* 10 are displayed.

The quality drop when linking registrations via one intermediate image was assessed for both individual segmentations and fused segmentations. Using the leave-one-out approach a fixed image *A*_*i*_ and an intermediate image *A*_*k*_, *k* ≠ *i* was selected. The other atlas images were in turn used as moving images *A*_*j*_, *j* ≠ *i*, *k*, thus for *N* = 10 a total of 10 ⋅ 9 ⋅ 8 = 720 combinations of registrations were possible. The segmentations from the moving images were transformed using the composed transformation *T*_*i* ← *I*(1) ← *j*_ = *T*_*i* ← *k*_ ∘ *T*_*k* ← *j*_ applied to the labeled images *L*_*j*_ and results were compared with results using direct registrations *T*_*i* ← *j*_. Figure 1 demonstrates this process with *i =* 1 and *k =* 2 . The quality loss was then quantified by comparing the resulting *DSC* values with those acquired based on direct registrations.

As the segmentation quality is expected to decrease when using an increasing number of intermediate images, we also investigated the quality change for a chain of linked registrations, i.e. use of

2 $$ \begin{array}{l}{T}_{j\leftarrow I(1)\leftarrow i}={T}_{j\leftarrow k}\circ {T}_{k\leftarrow i}\\ {}{T}_{j\leftarrow I(2)\leftarrow i}={T}_{j\leftarrow k}\circ {T}_{k\leftarrow m}\circ {T}_{m\leftarrow i}\\ {}\vdots \end{array} $$

up to the maximum possible number of combinations permitted by our atlas material, which yields a maximum of nine transformations. As the number of possible combinations quickly becomes so large that it is not realistic to calculate all of them, we sampled randomly the combinations used for evaluation.

### Label fusion

For the fused segmentation we only investigate linking two deformable registrations. Here, the label images *L*_*j*_ are transformed using *T*_*j* ← *I*(1) ← *i*_, *j* ≠ *i*, *k* and the label image belonging to the intermediate image, *L*_*k*_, is transformed using the direct registration *T*_*j* ← *k*_. This gives in total 90 test cases using the leave-one-out strategy. All registered label images were converted to one binary image *B*_*j*_ per structure. The multi-atlas fusions of segmentations were then created using weighted averaging of the structure’s distance maps. A signed distance map *D*_*j*_ for a binary image *B*_*j*_ is defined as the minimum Euclidean distance to the border voxels ∂*B*_*j*_ of the structure for all voxels in the image, i.e.

3 $$ {D}_j\left(x,y,z\right)=\underset{\left({x}_b,{y}_b,{z}_b\right)\in \partial {B}_j}{ \min}\sqrt{{\left(x-{x}_b\right)}^2+{\left(y-{y}_b\right)}^2+{\left(z-{z}_b\right)}^2} $$

Border voxels were defined as voxels that were 6-connected, i.e. sharing one side, to any voxels not part of the object. Distances inside the object were set to negative values. The resulting, fused segmentation is given by the iso-level zero of the fused distance map given by

4 $$ {D}_{\mathrm{fus}}=\frac{{\displaystyle \sum_j{w}_j{D}_j\left(x,y,z\right)}}{{\displaystyle \sum_j{w}_j}} $$

with probabilistic weights [10]

5 $$ {w}_i=\frac{1}{2}\left(1-\mathrm{e}\mathrm{r}\mathrm{f}\left(\frac{1}{\sqrt{2}}\frac{k}{s}\left(SI{M}_{\mathrm{best}}-SI{M}_i\right)\right)\right) $$

where *SIM*_*i*_ is the image similarity achieved for registration *i*, *SIM*_best_ the best similarity of all *i*, and *k* and *s* are control parameters. The control parameters could in principle be determined by linear regression of the segmentation quality versus image similarity, where *k* is the slope of the linear regression line and *s* is the standard deviation of the residuals of *DSC* to the linear regression line. In this work we have instead chosen to determine *k/s* from an overall optimization of the fusion results. For this process, the slope *θ* of a regression line was varied between -89 and +89 degrees in steps of 1 degree and the parameter *k*/*s* is then calculated by

6 $$ \frac{k}{s}=\frac{\sqrt{N} \tan \theta }{\sqrt{s{s}_{yy}+s{s}_{xx}{ \tan}^2\theta -2s{s}_{xy} \tan \theta }} $$

using $$ s{s}_{SIM}={\displaystyle \sum_i{\left(SI{M}_i-\overline{SIM}\right)}^2},s{s}_{SIM,DSC}={\displaystyle \sum_i\left(SI{M}_i-\overline{SIM}\right)\left(DS{C}_i-\overline{DSC}\right)}, $$

$$ s{s}_{DSC}={\displaystyle \sum_i{\left(DS{C}_i-\overline{DSC}\right)}^2}\kern0.5em \mathrm{and}\kern0.5em \overline{SIM}={\displaystyle \sum_i{\scriptscriptstyle \frac{SI{M}_i}{N}}},\overline{DSC}={\displaystyle \sum_i{\scriptscriptstyle \frac{DS{C}_i}{N}}}. $$

The normalized cross correlation (*NCC*) was used to calculate the image similarity measure

7 $$ SIM\left(F,M\right)=\frac{{\displaystyle \sum_{i,j,k}\left(F\left({x}_i,{y}_j,{z}_k\right)-\overline{F}\right)\left(M\left({x}_i,{y}_j,{z}_k\right)-\overline{M}\right)}}{\sqrt{{\displaystyle \sum_{i,j,k}\left(F\left({x}_i,{y}_j,{z}_k\right)-\overline{F}\right){\displaystyle \sum_{i,j,k}\left(M\left({x}_i,{y}_j,{z}_k\right)-\overline{M}\right)}}}}, $$

where the bars over *F* and *M* indicate arithmetic means of image intensities. The image similarity was calculated per structure over regions *C* defined by different cut-off distances *r* as

8 $$ D\left(x,y,z\right)\le r,\ \left(x,y,z\right)\in C. $$

Preliminary investigations of a value of *r* was performed by varying it from 0 to 50 mm in steps of 10 mm. For the mandible, with a well-defined border, a value of 0 mm was selected whereas for the other structures a value of 10 mm yielded the best results.

### Selection of intermediate image for linked registrations

The selection of the image to use as intermediate image for registration linkage might be of importance for the final result. One selection strategy would be to register a larger set of images using fast affine registrations and to use the resulting image similarities for ranking, as more similar images should be more suitable as atlases. By comparing results using this strategy to average results using all available intermediate images we noted that results were slightly improved by this strategy. To retain generality, we present results for decrease in segmentation quality using all available moving images as nodes in a leave-one-out fashion as previously described. For optimization of parameters for the probabilistic weighting algorithm based on image similarity information, the strategy of selecting the node with highest image similarity after affine registration was used.

## Results

**Figure 2:**
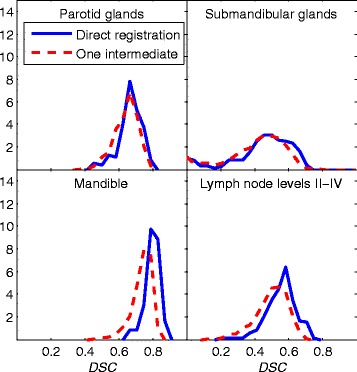
**The panels show the frequency distribution for the Dice similarity coefficient (** ***DSC*** **) for different structures based on transformations composed from two deformable registrations (dashed red) compared to direct registration results (solid blue).**

For both individual and fused segmentation results, there is a drop in *DSC* values for linked results using one intermediate image compared to direct results. The decrease in individual *DSC* values can be seen in Figure 2 as histogram plots for direct registration compared to linking of two deformable registrations. The mandible that is characterized by a distinct image gradient at the border show the largest drop in quality. Since Figure 2 displays results for structures which have not been fused by a label fusion algorithm, this figure also demonstrates the results for single atlas segmentation.

**Figure 3:**
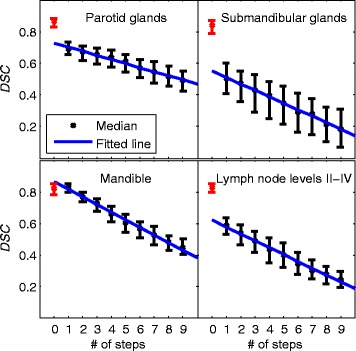
**The panels show the decrease of the Dice similarity coefficient (** ***DSC*** **) for structures subject to multiple linked deformable registration transformations.** The lines show the result of linear fits to the median values, and the whiskers extends from the 25th to 75th percentiles. The red symbols at zero links show the intra-user variability for redrawing respective structure two times (through editing of single atlas proposals). For the lymph node levels, only data for the union of levels I-VI was available and is shown instead.

When several registrations are linked, the median of the Dice similarity coefficient decreases approximately linearly with increasing number of linked registrations for all structures as shown in Figure 3. For comparison, *DSC* values for repeated segmentations of the same structures are shown for *l =* 0 registrations. For the *l =* 0 data points in Figure 3, previously unpublished data from [17] was used where one manual segmentation of a patient not included in the leave-one-out data was used as reference and compared to two manually improved (through editing) atlas-based segmentation proposals for the same patient. For the lymph node levels, unfortunately only the union of levels I-VI was available and is thus only an indication of the intra-user uncertainty of lymph node level segmentation as the segmentations in this work was using the unions of levels II-IV.

**Figure 4:**
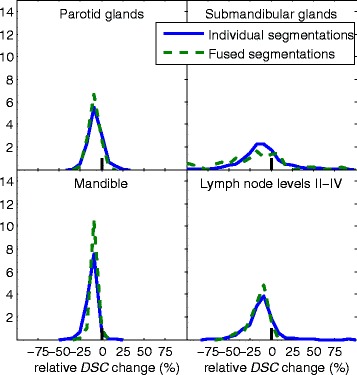
**Distributions of relative changes in Dice similarity coefficient (** ***DSC*** **) when linking two registrations compared to direct registration results.** Fused segmentations (green dashed lines) and individual segmentation results (blue lines). Different panels show different structures

**Figure 5:**
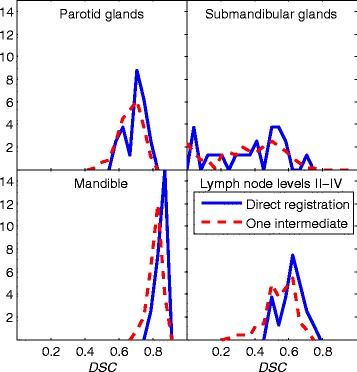
**Plots of the distribution of the Dice similarity coefficient (** ***DSC*** **) for fused structures and transformations composed from two deformable registrations compared to direct registration results.** Different panels show results for different structures.

**Figure 6:**
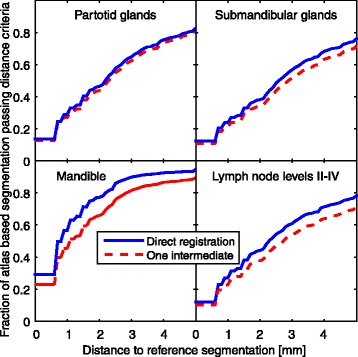
**Plots of the fraction of surface voxels within a given distance to the corresponding reference segmentation for fused segmentations.**

In Figure 4 the relative *DSC* change for the individual and fused segmentations are presented. Notably, for the least successful structures, the spread is very large, indicating that the spread of the locations of the segmentation results is larger and that some composed results actually yield better results than the corresponding direct registration. The distribution of segmentation quality for composed and direct segmentation using label fusion are shown in Figure 5 and the *fMAD* measure as a function of closest distance to the reference is presented for the structures in Figure 6.

**Figure 7:**
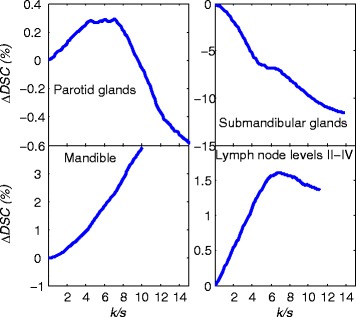
**Mean relative** ***DSC*** **change for the 10 patients when varying parameter** ***k*** **/** ***s*** **for weight calculations for probabilistic weighting, see equation** **5** **for an explanation of** ***k*** **and** ***s*** **.**

Results from the optimization of the *k*/*s* parameter for probabilistic weighting are shown in Figure 7 as relative change of mean *DSC* values. We see that the use of probabilistic weighting can improve results from the equally weighted segmentations, where *k*/*s* is zero. It is realized that for some cases, allocating higher weights to structures from more similar images actually decrease end results. By focusing on cases when end results are increased, we note that the mean DSC result improves further. As can be seen in Figure 7, reasonable values for *k*/*s* are 5-10 for all structures and that in general, higher *k*/*s* values should be used for structures with more clearly defined borders.

The relative reductions in median *DSC* for indirect compared to direct registrations for individual and fused segmentations can be seen in Table 1. The statistically significant decreases of median *DSC* for fused segmentations are in the range -2.8% to -8.4%.

**Table 1:** **Median relative change of** ***DSC*** **when using one linked registration compared to direct registration results**

	**Median relative change (%)**	
**Structure**	**Individual segmentations**	**Fused segmentations**
Parotid glands	−3.2 (-7.8, 1.9)*	−4.3 (-8.3, -0.7)*
Submandibular glands	−7.0 (-20, 4.4)*	−9.5 (-37, 11)
Mandible	−6.5 (-10, -3.7)*	−2.8 (-5.4, -1.3)*
Lymph node levels II-IV	−7.6 (-16, -1.0)*	−8.4 (-17, -3.1)*

In parenthesis the 25^th^ and 75^th^ percentiles are shown. Differences marked with (*) yielded a p-value <0.05 for a Wilcoxon rank-sum test.

The average total calculation times, including time for affine and deformable registrations as well as composition of transformations and label fusions are presented in Table 2. For a full atlas-based segmentation, this includes all 9 registrations as well as label fusion, and for segmentation using linked registrations, this includes one registration and 8 compositions of transformation through one atlas node. The label fusion time is approximately the same for both methods, and is added to both methods to report the total time for the segmentations.

**Table 2:** **Average time for full multi-atlas based segmentation including 9 affine and deformable registrations and label fusions**

	**Segmentation time (minutes and seconds)**	
	**Full multi-atlas registration**	**Linked registration**
Average time	35 minutes 32 s	9 minutes 59 s
Standard deviation	4 minutes 18 s	1 minute 43 s

In comparison, the time for segmentation using linked registrations, including one affine and deformable registration, composition of transformations and label fusion. The difference was found to be statistically significant (p < < 0.01 for a *t*-test).

## Discussion and conclusions

Transferring segmentations by composition of pre-calculated registration decrease the quality of registrations and atlas-based segmentations. However, our results demonstrate a moderate drop in segmentation quality while saving large amounts of calculation time, indicating that linking registrations is a feasible way of using multi-atlas registrations in a clinical setting. Registration times depend on actual methods and implementations, in this work the mean wall clock time for all registrations were 3 minutes and 50 seconds with a range from 3 minutes and 17 seconds to 4 minutes and 53 seconds. Compared to a full multi-atlas segmentation, the segmentation time was reduced to less than one third by using the linked registration method.

For the structure with the highest resulting *DSC*, the medulla, a larger drop in segmentation quality suggests that single atlas registration results, as can be seen in Figure 2, could be sufficient for this case.

In Figure 6 it can be noted that the average distance from the segmentation proposal to the reference segmentation is increased by linking registrations. The *fMAD* value gives an indication of the amount of editing needed before the structure is clinically acceptable. For the parotid glands, the quality decrease introduced by linking registrations, as measured by *fMAD,* is negligible. For the submandibular glands, there is a decrease of segmentation quality. Since manual re-contouring of the entire structures only takes a few minutes per structure, if only parotid glands and submandibular glands are to be segmented, the calculation time even for segmentations using linked registration will be at the order of complete manual re-contouring. However, if lymph node levels are part of the segmentation task for the creation of a treatment plan, the atlas-based segmentation method will yield segmentation proposals also for the glands with almost no additional calculation time. If the proposals for linked registrations will give shorter manual editing times compared to re-contouring remains to be investigated. For the lymph node levels that require a long manual contouring time, the significant time saving from using linked registrations compared to direct atlas-based segmentation will most likely lead to a reduction in total segmentation time.

The available atlases were based on images of patients with head-and-neck tumors that perturbed the normal anatomy. Since image registration is a more difficult problem when the images to be registered are less similar, selecting an atlas with a lower similarity to the current patient is likely to lead to a lower quality of the resulting segmentation. This will in turn lead to a larger spread in segmentation result based on the magnitude of the perturbation both in the patient selected through the leave-one-out process and the atlas selected as the node in the linking process. This can also explain why sometimes individual segmentations created by linked registrations have higher quality than direct segmentation results, for example for the submandibular glands.

We note from Figure 3 that the segmentation quality decrease is approximately linear to the number of links in the composed transformations. Segmentation quality for multi-atlas segmentation depends both on registration accuracy as well as the precision of the manual atlas segmentations. We hypothesize that extrapolation to zero registrations could provide observer independent information about the quality of the registrations and the limitations in precisions from the consensus protocol. If the registration accuracy is sufficient, the slope of the linear fit would be small and extrapolation to zero would indicate a limit in segmentation precision.

When label fusion is used, a similar reduction in segmentation quality as for the individual segmentations are seen. However, variance of the relative *DSC* change is decreased, as can be seen in Figure 5. This also means that the increase of segmentation quality that is occasionally seen for the individual results disappears, however to choose these improved segmentations is not trivial.

Time savings using a linked registration method compared to direct registrations are inversely proportional to the number of registrations used in the linking process. The additional overhead is simply the composition of the transforms, which can be implemented in different ways. If the transformations are pre-saved as full deformation fields, a simple linear interpolation per linking is the only additional over-head, which is very small compared to a complete registration. We compared segmentation results for composing registrations through successive linear interpolations with composition through the explicit B-spline transformations, with negligible differences in segmentation qualities.

## Consent

All the data used in this study were anonymized. The study design was approved by the ethical review board without the need for written informed consent.
